# ERCC1-expressing circulating tumor cells as a potential diagnostic tool for monitoring response to platinum-based chemotherapy and for predicting post-therapeutic outcome of ovarian cancer

**DOI:** 10.18632/oncotarget.13286

**Published:** 2016-11-11

**Authors:** Issam Chebouti, Jan Dominik Kuhlmann, Paul Buderath, Stephan Weber, Pauline Wimberger, Yvonne Bokeloh, Siegfried Hauch, Rainer Kimmig, Sabine Kasimir-Bauer

**Affiliations:** ^1^ Department of Gynecology and Obstetrics, University Hospital Essen, Essen, Germany; ^2^ Department of Gynecology and Obstetrics, Medical Faculty and University Hospital Carl Gustav Carus, Technische Universität Dresden, Dresden, Germany; ^3^ ACOMED Statistik, Department of Biostatics, Magdeburg, Germany; ^4^ QIAGEN Hannover GmbH, Langenhagen, Germany; ^5^ National Center for Tumor Diseases (NCT), Partner Site Dresden, Dresden, Germany; ^6^ German Cancer Consortium (DKTK), Dresden and German Cancer Research Center (DKFZ), Heidelberg, Germany

**Keywords:** circulating tumor cells, ERCC1, platinum-resistance, ovarian cancer, prognosis

## Abstract

**Background:**

We recently showed that the presence of ERCC1^+^CTCs is an independent predictive biomarker for platinum-resistance and poor prognosis of ovarian cancer. The goal of our current research was to determine how the auxiliary assessment of ERCC1-transcripts influences overall CTC-detection rate. We extended this investigation from an initially predictive setting to paired pre- and post-therapeutic blood analysis in order to see, whether ERCC1^+^CTCs dynamics mirror response to chemotherapy.

**Methods:**

65 Paired blood samples (10ml) of primary ovarian cancer patients at primary diagnosis and after chemotherapy were studied for CTCs with the AdnaTest *Ovarian Cancer* (QIAGEN Hannover GmbH). We analyzed the tumor-associated transcripts EpCAM, MUC-1 and CA-125. ERCC1-transcripts were investigated in a separate approach by singleplex RT-PCR.

**RESULTS:**

Auxiliary assessment of ERCC1-transcripts enhanced the overall CTC-detection rate up to 17%. ERCC1^+^CTCs (defined as positive for one of the AdnaTest markers plus ERCC1-positivity) were detected in 15% of patients at primary diagnosis and in 12% after chemotherapy. The presence of ERCC1^+^CTCs after chemotherapy correlated with platinum-resistance (P=0.01), reduced PFS (P=0.0293) and OS (P=0.0008) and their persistence indicated poor post-therapeutic outcome (PFS: P=0.005; OS: P=0.0058). Interestingly, the assessment of ERCC1-transcripts alone was sufficient for the detection of prognostic relevant ERCC1-expressing CTCs.

**Conclusion:**

Auxiliary assessment of ERCC1-transcripts expands the phenotypic spectrum of CTC detection and defines an additional overlapping fraction of ERCC1-expressing CTCs, which are potentially selected by platinum-based chemotherapy. Specifically, we suggest that ERCC1^+^CTCs could additionally be useful as a surrogate for monitoring platinum-based chemotherapy and to assess the post-therapeutic outcome of ovarian cancer.

## INTRODUCTION

Epithelial ovarian cancer is the fifth leading cause of cancer death of women in Europe and the United States and the second most common gynecological malignancy [1]. Most cases are diagnosed in advanced stages and, although response rates to chemotherapy reach up to 80%, the majority of patients cannot be cured. Standard treatment of advanced ovarian cancer is primary surgery aiming at complete macroscopic tumor resection followed by platinum- and paclitaxel-based chemotherapy, which has been shown to prolong progression free survival (PFS) as well as overall survival (OS) [2]. Postoperative residual tumor is one of the most important prognostic factors in advanced ovarian cancer [3–5]. However, despite advances in treatment, more than half of all patients will experience recurrence, resulting in poor overall survival [6].

Importantly, resistance to platinum-based chemotherapy, which can be caused by e.g. enhanced DNA-repair capacity of tumor cells, occurs in about 15-20% of patients and constitutes one of the most recognized clinical challenges for ovarian cancer [7]. The nucleotide excision repair (NER) pathway is a key pathway involved in mediating resistance or sensitivity to platinum-based chemotherapeutic agents. The excision repair cross-complementation group 1 (ERCC1) protein plays a key role in NER. It dimerizes with xeroderma pigmentosum complementation group F (known as ERCC4) and mediates the excision of DNA-platinum adducts, typically induced by platinum-based chemotherapy [8]. ERCC1-expression has been extensively studied in primary tumor tissue of several cancer entities, including ovarian cancer, and has been proposed as a potential predictor for response to platinum-based chemotherapy. However, this concept has been controversial, particularly in the context of immunohistochemical ERCC1-detection, and has not been implemented into clinical routine so far [9–20]. Taking into account that primary tumor tissue is typically available only at primary diagnosis, it would be valuable to establish a non-invasive blood-based biomarker for stratifying response to platinum-based chemotherapy at primary diagnosis and for guiding individualized therapy decisions in the future. We recently showed that the presence of ERCC1^+^CTCs (circulating tumor cells) at primary diagnosis of ovarian cancer, a potentially platinum-resistant CTC-subgroup, is an independent predictive biomarker for primary platinum-resistance and poor prognosis of ovarian cancer [21].

We now explored in more detail, in how far the auxiliary assessment of ERCC1-transcripts influences overall CTC-detection rate and whether this molecular marker may improve the phenotypic range of CTC-detection by the AdnaTest *Ovarian Cancer* platform. We essentially extended this investigation from an initially predictive setting to paired pre- and post-therapeutic blood analysis and explored clinical relevance of ERCC1^+^CTC dynamics in response to platinum-based chemotherapy.

## RESULTS

### Influence of auxiliary ERCC1-transcript assessment on the CTC-detection rate

We previously have demonstrated that ERCC1 extends clinical information of CTCs as a prognostic biomarker to the prediction of platinum-resistance at primary diagnosis of ovarian cancer [21]. We now explored in more detail how additional assessment of ERCC1 influences the overall detection rate of CTCs in 65 paired pre-operative and post-chemotherapeutic blood samples from ovarian cancer patients. First we assessed the marker transcripts according to the AdnaTest *Ovarian Cancer* in its previous configuration. CTC-positivity of this assay was indicated by the detection of at least one of the transcripts EpCAM, MUC-1 or CA-125, herein referred to as “AdnaTest^+^”. Furthermore, we now considered ERCC1-transcripts as an additional marker for CTC-detection.

**Figure 1:**
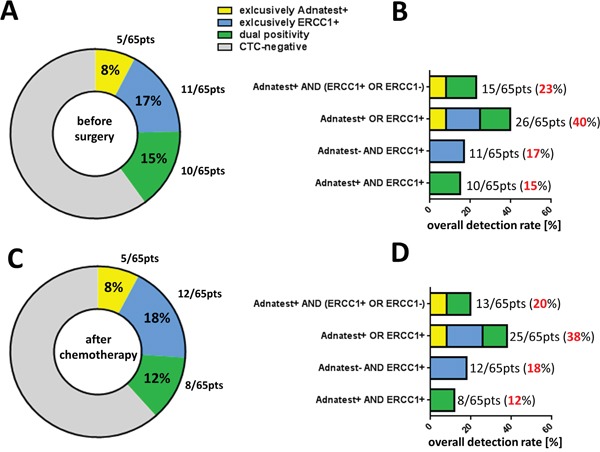
Influence of auxiliary ERCC1-transcript assessment on CTC-detection rate **A**. The pie chart shows the different CTC-types and their relative proportions among the studied ovarian cancer patients before surgery (n=65). Percentages indicate the proportion of patients with exclusively-Adnatest-positivity (yellow), exclusively-ERCC1-positivity (blue), dual-positivity for Adnatest/ERCC1 (green) and CTC-negative patients (grey). **B**. The stacked bar chart summarizes four CTC-definition criteria, considering ERCC1 as additional transcript marker and shows, how this is translated into different overall CTC-detection rates. **C+D**. These illustrations depict the same type of analysis as reported above, however refer to paired blood samples analyzed after platinum-based chemotherapy (n=65). In all figures, absolute patient numbers in each subgroup are indicated.

Figure 1A summarizes the detected CTC-types and shows their relative proportions among the studied ovarian cancer patients. In 8% of patients AdnaTest-positivity was exclusively observed. In 17% we detected exclusively ERCC1-positive CTCs and in 15% we observed dual positivity for the AdnaTest and ERCC1. Subsequently, we were interested in how auxiliary assessment of ERCC1 influences the overall detection rate of CTCs in ovarian cancer. Therefore, we compared overall CTC-detection rates across several defining criteria for “CTC-positivity”, with the presence of ERCC1-transcripts as an additional alternative criterion or as obligatory requirement, respectively (Figure 1B): We observed a CTC-detection rate of 23% before surgery, comprised of patients with only AdnaTest positivity and dual AdnaTest/ERCC1-positive patients. Detection rates were substantially increased up to 40% if ERCC1 was considered as a further alternative marker for CTC-positivity (AdnaTest^+^ OR ERCC1^+^). Since this CTC-definition now comprises a further subgroup of patients with exclusively ERCC1-expressing CTCs. This subgroup alone can be detected with an overall detection rate of 17% (AdnaTest^−^ AND ERCC1^+^). Lastly, according to a more stringent definition of combined positivity (AdnaTest^+^ AND ERCC1^+^), overall detection decreased to 15%.

After platinum-based chemotherapy the proportion of CTC-subtypes and their overall detection rates among the above mentioned CTC-definition criteria were grossly comparable with those found before therapy (Figure 1C+1D).

### ERCC1^+^CTCs predict post therapeutic outcome

The median follow up time for PFS was 37 months (range 4-120 months) resulting in 36 (55%) relapses while 28 patients (43%) presented with no relapse. After a median follow-up time of 45 months (range 11-117 months) for OS, 42 patients (65%) were still alive and 23 patients (35%) had died (Table 1).

**Table 1:** Patient characteristics at the time of primary diagnosis

Total	65
Age	median 61 years, (27-92)
FIGO stage	
I-II	11 (17%)
III	41 (63%)
IV	13 (20%)
Nodal status	
N_o_	24 (37%)
N_1_	28 (43%)
N_x_	13 (20%)
Grading	
I-II	28 (43%)
III	37 (57%)
Unknown	0 (0%)
Residual tumor	
Macroscopic	
Complete resection	38 (58%)
Any residual tumor	27 (42%)
Histologic type	
Serous	52 (80%)
Mucinous	9 (14%)
Other	4 (6%)
Survival	
PFS^1^	median 37 months, (4-120 months)
OS^2^	median 45 months, (11-117 months)
Alive	42 (65%)
Dead	23 (35%)
Unknown	0 (0%)
Recurrence	
No relapse	28 (43%)
Relapse	36 (55%)
Unknown	1 (2%)

^1^PFS: progression-free survival, ^2^OS: overall survival

**Figure 2:**
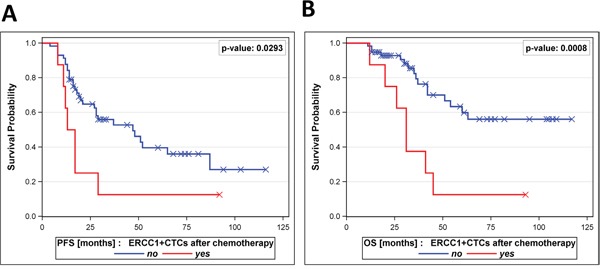
Prognostic relevance of ERCC1^+^CTCs after chemotherapy A patient was considered positive for ERCC1^+^CTCs if at least one of the AdnaTest transcript markers (EpCAM, MUC-1 or CA-125) was detected, in addition to ERCC1-positivity. The Kaplan-Meier analysis shows **A**. progression-free survival and B. overall survival of patients with detectable ERCC1^+^CTCs after platinum-based chemotherapy (bottom curves) in comparison to patients with non-detectable ERCC1^+^CTCs (top curves).

We first explored the clinical relevance of ERCC1-expressing CTCs in post-therapeutic blood samples. For this purpose, in accordance with our previous publication [21], we primarily focused on the most stringent definition of ERCC1-expressing CTCs, which is based on the previous AdnaTest markers (EpCAM, MUC-1, CA-125) and encludes ERCC1-positivity as an additional obligatory requirement (AdnaTest^+^ AND ERCC1^+^). This cell population is from now on referred to as ERCC1^+^CTCs. The presence of post-therapeutic ERCC1^+^CTCs significantly correlated with decreased PFS (p=0.0293) and OS (p=0.0008, Figure 2A+2B). Furthermore, the presence of ERCC1^+^CTCs after chemotherapy correlated with primary platinum-resistance (p=0.01, data not shown).

### ERCC1^+^CTC dynamics mirror response to platinum-based chemotherapy

**Figure 3:**
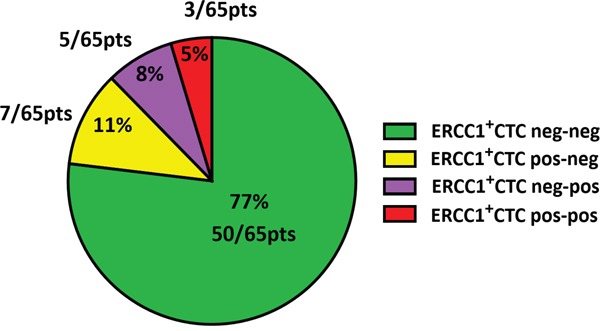
Dynamics of ERCC1^+^CTCs in the course of platinum-based chemotherapy A patient was considered positive for ERCC1^+^CTCs if at least one of the Adnatest transcript markers (EpCAM, MUC-1 or CA-125) was detected, in addition to ERCC1-positivity. The pie chart shows a stratification of the study cohort (n=65) into different subgroups, according to the dynamics of ERCC1^+^CTCs before surgery and after chemotherapy. Besides the group of patients, who were negative for ERCC1^+^CTCs throughout (ERCC1^+^CTCs neg-neg), we observed patients, who became negative after chemotherapy (ERCC1^+^CTCs pos-neg), patients with newly acquired positivity after chemotherapy (ERCC1^+^CTCs neg-pos) or persistently positive patients (ERCC1^+^CTCs pos-pos). Percentages and absolute patient numbers are indicated.

We were further interested in how the levels of ERCC1^+^CTCs in our patients changed in response to platinum-based chemotherapy. A stratification of our study patients according to “ERCC1^+^CTCs dynamic subgroups” is presented in Figure 3. The majority of patients were negative for ERCC1^+^CTCs throughout (77%, “neg-neg”) treatment. In 11% of patients we observed ERCC1^+^CTCs before surgery, which disappeared after platinum-based chemotherapy (“pos-neg”). Moreover, 8% of patients were initially negative and ERCC1^+^CTCs newly appeared after therapy (“neg-pos”). Finally, in 5% of patients, persistent ERCC1^+^CTCs were observed before surgery and after chemotherapy (“pos-pos”).

**Figure 4:**
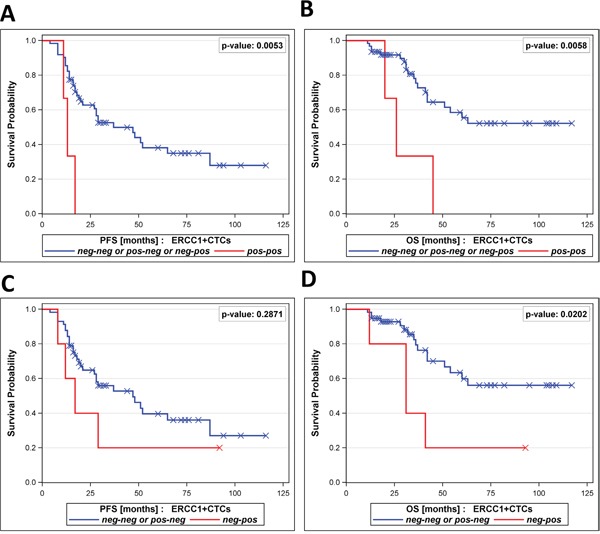
Prognostic relevance of persistent ERCC1^+^CTCs The Kaplan-Meier plots show **A**. progression-free survival and **B**. overall survival of patients with persistent positivity for ERCC1^+^CTCs in their blood (ERCC1^+^CTC pos-pos, bottom curves), in comparison to all other dynamic subgroups together (ERCC1^+^CTC pos-neg / neg-pos / neg-neg, top curves). Moreover, Kaplan-Meier plots show **C**. progression-free survival and **D**. overall survival of patients with newly acquired positivity for ERCC1^+^CTCs (ERCC1+CTC neg-pos, bottom curves), in comparison to the dynamic subgroups ERCC1^+^CTC pos-neg and ERCC1^+^CTC neg-neg, together (top curves).

Interestingly, patients with persistent positivity for ERCC1^+^CTCs before surgery and after chemotherapy (ERCC1^+^CTC “pos-pos”) had a very poor PFS (p=0.0053) and OS (p=0.0058, Figure 4A+4B) compared to all other dynamic subgroups together (“neg-neg” or “pos-neg” and “neg-pos”). Furthermore, we observed the trend that patients with newly acquired ERCC1^+^CTCs after chemotherapy (“neg-pos”) had a shorter PFS by trend (p=0.2871) and a significantly shorter OS (p=0.0202) than the “neg-neg” group and the “pos-neg” group together (Figure 4C+4D).

### The assessment of ERCC1-transcripts alone is a surrogate for the detection of prognostically relevant CTCs

**Figure 5:**
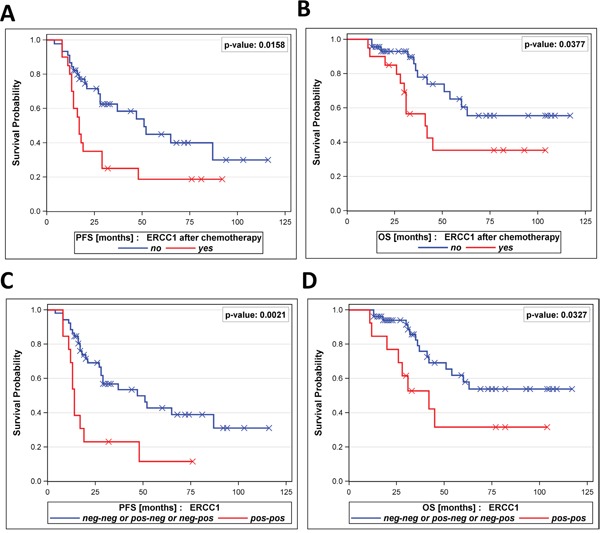
Prognostic relevance of ERCC1-transcripts alone This analysis refers to the prognostic relevance of ERCC1-transcripts alone, irrespectively of the Adnatest transcript markers EpCAM, MUC-1 or CA-125. The Kaplan-Meier plots show **A**. progression-free survival and **B**. overall survival of patients with ERCC1-positivity after platinum-based chemotherapy (bottom curves) in comparison to patients with non-detectable ERCC1-transcripts (top curves). Moreover, Kaplan-Meier plots show **C**. progression-free survival and **D**. overall survival of patients with persistent positivity for ERCC1-transcripts (ERCC1^+^ pos-pos, bottom curves) in comparison to all other dynamic subgroups together (ERCC1^+^ pos-neg / neg-pos / neg-neg, top curves).

We were interested in how prognostic information as described above was retained when ERCC1-transcripts alone were assessed. Therefore, we exclusively focused on ERCC1-transcript expression, irrespectively of EpCAM, MUC-1 or CA-125 positivity and re-performed survival analysis. The presence of post-therapeutic ERCC1-transcript positivity alone indicated reduced PFS (p=0.0158) and OS (p=0.0377, Figure 5A+5B). Once more, a stratification of our study patients according to “ERCC1 dynamic subgroups” was performed and is presented in Supplementary Figure S1. The majority of patients were negative for ERCC1-transcritps throughout treatment (57%, “neg-neg”). In 12% of patients we observed ERCC1-positivity before surgery which disappeared after platinum-based chemotherapy (“pos-neg”). Moreover, 11% of patients were initially negative and ERCC1^+^CTCs newly appeared after therapy (“neg-pos”). Finally, in 20% of patients, persistent ERCC1^+^CTCs were observed before surgery and after chemotherapy (“pos-pos”). More interestingly, as already reported for ERCC1^+^CTCs, the ERCC1 “pos-pos” subgroup also had a significantly decreased PFS (p=0.0021) and OS (p=0.0327), compared to all other dynamic subgroups together (Figure 5C+5D). Of note is that the statistical significance level of these findings was generally lower compared to the prognostic relevance of ERCC1^+^CTCs, which referred to combined CTC- and ERCC1-positivity.

The number of patients at risk in each of the subgroups shown in Figures 2, 4 and 5 are documented in Supplementary Table S1.

## DISCUSSION

In the present study we demonstrate that the additional assessment of ERCC1-transcripts enhanced overall CTC detection rate in ovarian cancer patients. It also defines an additional overlapping fraction of ERCC1-expressing CTCs, which are potentially selected by platinum-based chemotherapy. We also describe that the assessment of CTC-derived ERCC1-transcripts alone was almost equivalently sufficient in order to detect ERCC1-expressing prognostic relevant CTCs. We further showed that the presence of ERCC1^+^CTCs after chemotherapy correlated with post-therapeutic outcome of ovarian cancer and, particularly, dynamics of ERCC1^+^CTCs mirrored response to platinum-based chemotherapy.

We have already demonstrated that a) additional detection of ERCC1-transcripts extented clinical value of CTCs from a prognostic biomarker to an independent predictor of platinum-resistance at primary diagnosis of ovarian cancer and b) ERCC1^+^CTCs may constitute a distinct subgroup of CTCs with a potentially platinum-resistant phenotype [21]. We now confirmed that auxiliary assessment of ERCC1-transcripts considerably expanded the phenotypic spectrum of CTC-detection in pre- and post-therapeutic blood samples and that ERCC1 obviously marks a definable CTC-phenotype with overlap to the CTC-population, as detected by the AdnaTest *Ovarian Cancer*. Therefore, in a considerable number of patients, CTC-derived ERCC1-expression was accompanied by co-expression of at least one of the standard markers for CTC-detection (EpCAM or MUC-1 or CA-125). Given the experimental setting of the AdnaTest, we cannot distinguish whether this co-expression was derived from CTCs actually co-expressing these markers on the same cell, or from separate CTC-populations which were concomitantly present in the “pool” of immunomagnetically enriched CTCs from a given blood sample. However, we also observed a minor subset of patients who were exclusively positive for ERCC1-transcripts. We suppose that these patients harbor epithelial-associated CTCs in their blood, which express EpCAM or MUC-1 antigens on their surface. These CTCs were captured by the AdnaTest selection procedure which targets EpCAM and MUC-1 surface epitopes. However EpCAM and MUC-1 transcripts seemed to be downregulated on the transcriptional level in these isolated CTCs. Discordances between protein and transcript expression profiles of a cell could be due to post-transcriptional modifications of messenger RNA or differences in the half-life time between messenger RNA and their corresponding proteins [22–24].

The broad heterogeneity of CTCs in the blood of cancer patients, including ovarian cancer, has already been indicated by several independent reports [25–27]. We may hypothesize that ERCC1-expressing CTCs play a dominant role during the course of the disease, which is corroborated by the fact that the rate of exclusively ERCC1-positive CTCs did not decrease after platinum-based chemotherapy. We have already shown that breast cancer patients with CTCs detected after neoadjuvant chemotherapy were associated with tumor stem cell characteristics and ERCC1-expression [28]. This may suggest a potential selection of this CTC-subset by chemotherapy. However, due to the fact that exclusively ERCC1-expressing CTCs rarely occurred in our patient cohort, we were not able to analyze prognostic significance of this particularly interesting CTC-subset in a statistically substantiated manner.

Independent of ERCC1 assessment, we previously confirmed the negative prognostic impact of CTCs before surgery and after chemotherapy, as detected by the AdnaTest *Ovarian Cancer* [21, 29]. Nevertheless, our recent and current results strongly support our perception that the auxiliary assessment of ERCC1-transcripts provides complementary clinical information. In addition, the auxiliary assessment of ERCC1-transcripts after chemotherapy alone, as well as their expression dynamics in pre- and post-therapeutic blood samples, was almost equivalently sufficient as surrogate for a CTC-population. This might be useful for predicting post-therapeutic outcome and for monitoring platinum-based chemotherapy. Given the additional strong prognostic impact of the standard AdnaTest marker transcripts MUC-1, EpCAM and CA-125 [21, 29] and considering that the statistical significance level slightly declined when only ERCC1-transcripts were assessed, a combined condition, which assumes ERCC1-positivity in addition to the detection of at least one of the AdnaTest markers (referred to as ERCC1^+^CTCs in our study), appears to be most favorable in terms of a blood-based prognostic biomarker.

So far, any functional characteristics of ERCC1-expressing CTCs in the blood of ovarian cancer patients are unknown. Since our study was performed exclusively from a “biomarker perspective”, we can only assume that ERCC1-(over)expressing CTCs in the blood may be characterized by an enhanced, preexisting or newly acquired capacity to resolve DNA-platinum-adducts, consequently bypassing cisplatin-mediated cytotoxicity and possibly converting to a molecular phenotype of “on-target” platinum-resistance [8]. This assumption is further supported by a recent investigation which directly analyzed the presence of DNA-platinum adducts in single CTCs of advanced non-small cell lung cancer (NSCLC) patients. In this context, it was suggested that the kinetics of these adducts in pre- and post- therapeutic blood samples could be a potential biomarker for response prediction and dose individualization of platinum-based chemotherapy [30]. Consecutively, ERCC1^+^CTCs may survive multiple cycles of chemotherapy and, in line with the fact that metastasis-initiating cells can be present among CTCs in the blood [31], persistent ERCC1^+^CTCs with a platinum-resistant phenotype could have the potential to initiate recurrence, resulting in poor clinical outcome. Taking into consideration that ERCC1^+^CTCs are strong prognostic factors in the post-therapeutic situation, particularly in case of persistent positivity, our data may also indicate that platinum-resistant ERCC1^+^CTCs could be directly selected upon platinum-based chemotherapy. Further functional studies will be necessary in order to prove this hypothesis. Another interesting question for future studies will be, how CTC-derived ERCC1-expression is related to EMT- and stem-like characteristics of CTCs. In this regard, we recently demonstrated that the negative prognostic impact of the presence and/or persistence of disseminated tumor cells in the bone marrow of ovarian cancer patients after platinum based chemotherapy was associated with stem cell character [32].

Conclusively, due to the limited number of patients, our study is explorative and hypothesis generating. Nevertheless, ERCC1 marks a subpopulation of CTCs which might be useful for monitoring platinum-based chemotherapy and for assessing post-therapeutic outcome of ovarian cancer patients. We provide rationale to validate clinical utility of ERCC1^+^CTCs among large multicenter clinical trials and to further elucidate their functional and tumor biological significance. Alternatively, patients with ERCC1*CTCs may profit from an early initiated and dose-intense maintenance therapy with e.g. Bevacizumab or PARP-inhibitors. Furthermore, this high risk patient group might be amenable to platinum-sensitizing therapies in the future, which are increasingly proposed in preclinical studies [33–35] and already ongoing clinical trials (NCT01164995). Using CTCs as liquid biopsy tool for individual therapy optimization, a multi-marker gene panel, compromising all CTC subgroups, will be useful to monitor patients during the course of the disease [36].

## PATIENTS AND METHODS

### Patient characteristics

The present study was conducted at the Department of Gynecology and Obstetrics at the University Hospital of Essen, Germany. A total of 65 patients diagnosed between 2006 and 2014 with histologically confirmed epithelial ovarian cancer were analyzed. Clinical characteristics of the patients are documented in Table 1. Informed written consent was obtained from all patients and the study was approved by the Local Ethics Committee (05-2870) and performed according to the declaration of Helsinki. Tumors were classified according to the WHO classification of tumors of the female genital tract. Grading was conducted using the grading system proposed by Silverberg [37] and tumor staging was classified according to the Fédération Internationale de Gynécology et d'Obstétrique [38]. The whole study population underwent primary radical surgery. Total abdominal hysterectomy, bilateral salpingo-oophorectomy, infragastric omentectomy, peritoneal stripping were performed. The most important aim of surgery was to achieve macroscopic complete tumor resection. Radical pelvic and para-aortic lymphadenectomy were only performed if macroscopic complete tumor resection was achieved intraperitoneally following actual guidelines. All patients received at least six cycles of carboplatinum AUC 5 and paclitaxel 175 mg/m^2^. Tumors were clinically defined as platinum-resistant if they recurred within six months after the completion of platinum-based chemotherapy.

### Enrichment and molecular characterization of CTCs

Peripheral blood (2×5 ml) from each patient was collected in EDTA tubes (Sarstedt & Co.) and processed within 4h for the enrichment of CTCs and subsequent expression analysis according to Adnatest *Ovarian Cancer* (QIAGEN, Hannover GmbH, Langenhagen, Germany). The test has been described in detail [21]. Briefly, CTCs were immunomagnetically selected using the AdnaTest *Ovarian Cancer Select* targeting epithelial cell adhesion molecule EpCAM (also known as GA733-2), Mucin-1, cell surface associated (MUC-1) and cell surface associated Mucin-16 (also known as CA-125). Subsequently, RNA was isolated and gene expression analysis was performed by reverse-transcription (RT) and multiplex RT-PCR detecting EpCAM, MUC-1, and CA-125 (AdnaTest *Ovarian Cancer Detect)*. ERCC1-transcripts were investigated in a separate approach by singleplex RT-PCR. β-actin served as an internal control and PCR-products were quantified on the Agilent Bioanalyzer as follows: Blood samples of 20 healthy donors and healthy donor blood samples spiked with two or five cells IGROV1 were analyzed using the AdnaTest *Ovarian Cancer Select/Detect* for overexpression of EpCAM, MUC-1 and CA-125. The resulting PCR fragments were analyzed with the Agilent Bioanalyzer and the resulting data were checked for sensitivity and specificity to be ≥ 90% applying a 0.15 ng/μl fragment concentration as a cut of value for each of the markers. For the required specificity of >90%, as defined in the test performance criteria, the resulting cut off value was defined as 0.15 ng/μl fragment concentration. At a cut-off value of 0.15 ng/μl specificity is 95% and the corresponding recovery rate is 80% for two cells and 100% for five cells, respectively.

ERCC1-positivity was defined by an amplicon concentration >0.2ng/μl. Sensitivity and specificity were evaluated in 20 healthy donors and 99 patients with primary ovarian cancer using ROC analysis. At a cut-off value of 0.17 ng/μl, 95% specificity is reached and the corresponding clinical sensitivity is 46.5% (Supplementary Figure S2).

Amplicons with the following sizes were generated: EpCAM: 396bp; MUC-1: 293bp; CA-125: 432bp; ERCC1: 366bp; and β-actin: 114bp.

### Statistical analysis

Survival curve plots and Hazard Ratio calculations were done using SAS (9.4). Survival intervals were screened from the time of CTC detection at first diagnosis to the time of clinical event (either death or first time of relapse) or last contact. Kaplan-Meier curves were assessed using the log-rank test to evaluate univariate significance of the binary grouping parameters. Fisher exact tests were performed to confirm significance. Survival curve plots and Hazard Ratio calculations were done using SAS (9.4).

## SUPPLEMENTARY MATERIALS FIGURES AND TABLES


