# Supplementary material for: ERCC1-expressing circulating tumor cells as a potential diagnostic tool for monitoring response to platinum-based chemotherapy and for predicting post-therapeutic outcome of ovarian cancer
Source: Oncotarget. 2016 Nov 11;8(15):24303–13. doi: 10.18632/oncotarget.13286 (PMC5421848; doi:10.18632/oncotarget.13286)
Supplement: Supplementary file 1 [file oncotarget-08-24303-s001.pdf]

ERCC1-expressing circulating tumor cells as a potential diagnostic tool for monitoring response to platinum-based chemotherapy and for predicting post-therapeutic outcome of ovarian cancer

SUPPLEMENTARY FIGURES AND TABLE

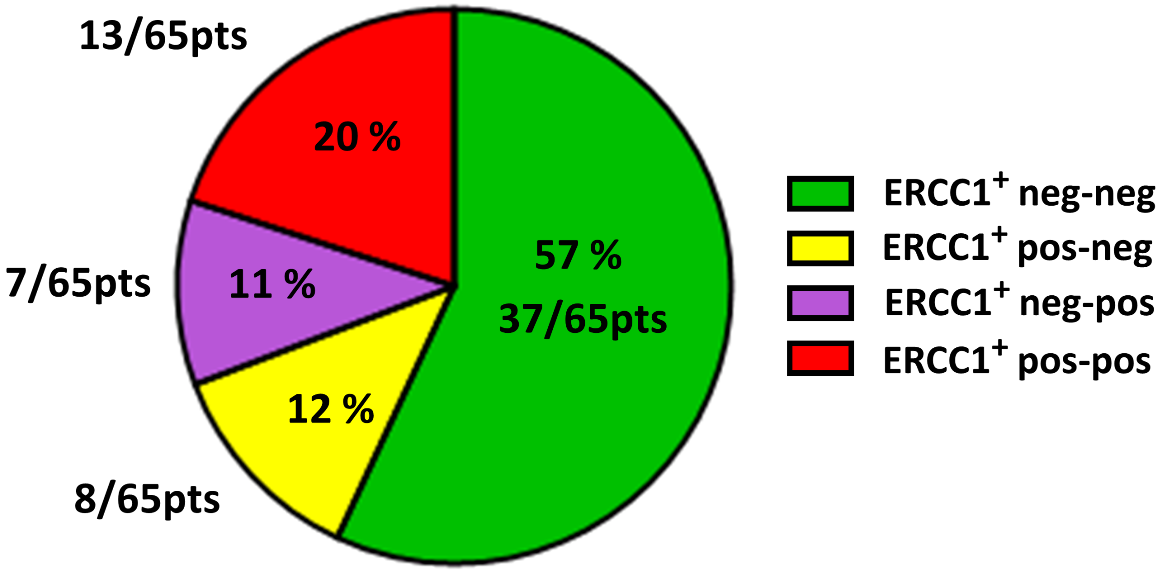

Supplementary Figure S1: Dynamics of ERCC1-positivity in the course of platinum-based chemotherapy. This analysis refers to the prognostic relevance of ERCC1-transcripts alone, irrespectively of the Adnatest transcript markers EpCAM, MUC-1 or CA-125. The pie chart shows a stratification of the study cohort (n=65) into different subgroups, according to the dynamics of ERCC1-positivity ($ERCC1^+$) before surgery and after chemotherapy. Beside the group of patients, who were negative for ERCC1 throughout treatment ($ERCC1^+$ neg-neg), we observed patients, who became negative after chemotherapy ($ERCC1^+$ pos-neg), patients with newly acquires positivity after chemotherapy ($ERCC1^+$ neg-pos) or persistently positive patients ($ERCC1^+$ pos-pos). Percentages and absolute patient numbers are indicated.

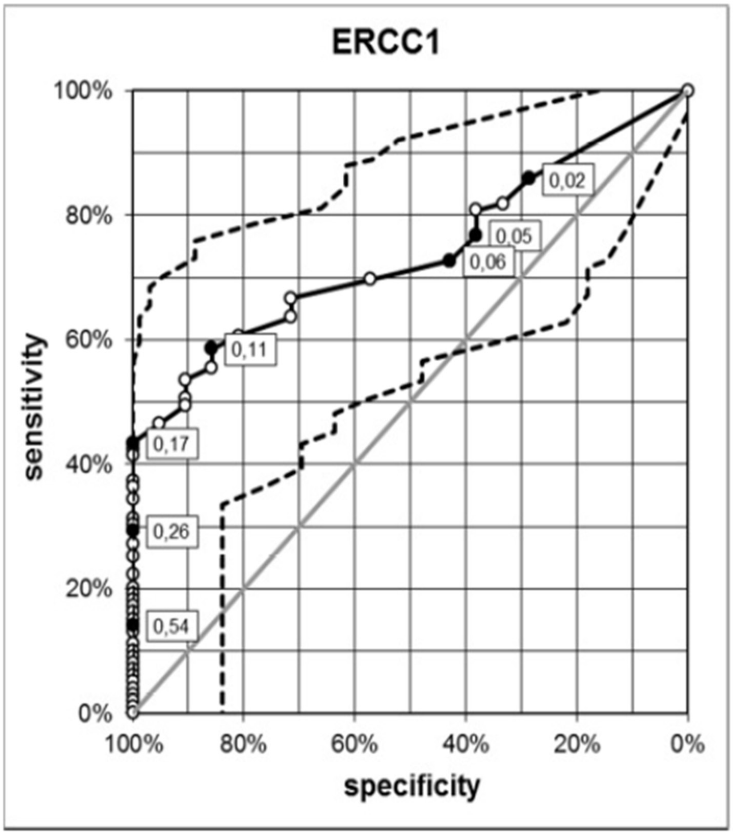

N (normal)	N (abnormal)
21	99
ROC CURVE	P1
AUC	0,7367
SE	0,0528
low. CI (0,95)	0,6483
upp. CI (0,95)	0,8130
SPEC	SENS
95%	46,5%
90%	53,5%
80%	60,6%
SENS	SPEC
95%	0,0%
90%	0,0%
80%	38,1%

Supplementary Figure S2: ROC analysis for the determination of ERCC1 sensitivity and specificity. Blood samples of 20 healthy donors and 99 patients with primary ovarian cancer were analyzed for CTCs with the AdnaTest *OvarianCancer* and for ERCC1 expression applying densitometric fagment quantification using the Agilent Bioanalyzer 2100. The resulting data were checked for sensitivity and specificity. A cut off value was determined using a ROC analysis. At a cut-off value of 0.17 ng/µl 95% specificity was reached and the corresponding clinical sensitivity was 46.5%. A slightly higher cut-off at 0.2 ng/µl was, however, chosen as kind of a security measure.

Supplementary Table S1: Univariate analysis of different CTC-subgroups and patient numbers at risk

			at risk (%)			p-value (log rank)
**1. PFS**	**0**	**25**	**50**	**75**	**100**	
**ERCC-1 after chemotherapy**						**0.0158**
no	45	25	12	4	2	
yes	20	7	3	3	0	
**ERCC-1**						
pos-pos	13	3	1	1	0	**0.0021**
neg-neg or pos-neg or neg-pos	52	29	14	6	2	
**ERCC-1$^+$CTCs after chemotherapy**						**0.0293**
no	57	30	14	6	2	
yes	8	2	1	1	0	
**ERCC-1$^+$CTCs**						
pos-pos	3	0	0	0	0	**0.0053**
neg-neg or pos-neg or neg-pos	62	32	15	7	2	
**ERCC-1$^+$CTCs**						
neg-pos	5	2	1	1	0	0.2871
neg-neg or pos-neg	57	30	14	6	2	
			**at risk (%)**			**p-value(log rank)**
**2. OS**	**0**	**25**	**50**	**75**	**100**	
**ERCC-1 after chemotherapy**						**0.0377**
no	45	31	17	8	5	
yes	20	16	5	5	1	
**ERCC-1**						
pos-pos	13	10	3	3	1	**0.0327**
neg-neg or pos-neg or neg-pos	52	37	19	10	5	
**ERCC-1$^+$CTCs after chemotherapy**						**0.0008**
no	57	41	21	12	6	
yes	8	6	1	1	0	
**ERCC-1$^+$CTCs**						
pos-pos	3	2	0	0	0	**0.0058**
neg-neg or pos-neg or neg-pos	62	45	22	13	6	
**ERCC-1$^+$CTCs**						
neg-pos	5	4	1	1	0	**0.0202**
neg-neg or pos-neg	57	41	21	12	6	

The table summarizes univariate analysis according to the Kaplan-Meier curves (Figure 2, 4, 5). The absolute number of patients in each subgroup at a risk of 0%, 25%, 50%, 75% and 100% is indicated for each case.
